# Supplementary figures and images for: Perirenal adipose tissue contains a subpopulation of cold-inducible adipocytes derived from brown-to-white conversion
Source: eLife. 2024 Mar 12;13:RP93151. doi: 10.7554/eLife.93151 (PMC10932542; doi:10.7554/eLife.93151)

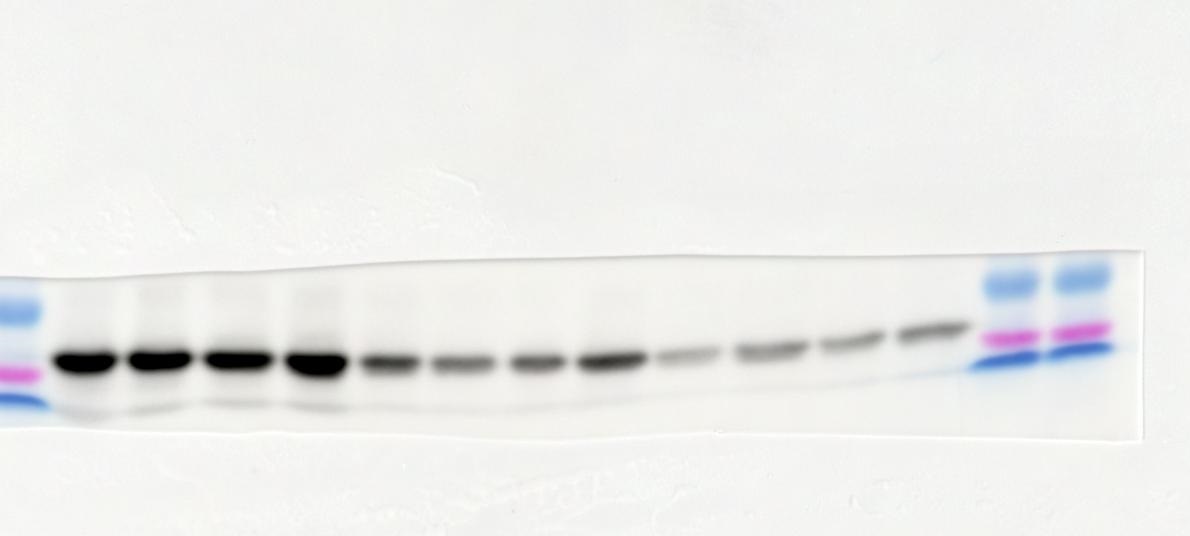

Supplement: Figure 4—source data 1. [file elife-93151-fig4-data1.zip › Figure 4 í¬ source data 1/iBAT UCP1.jpg]

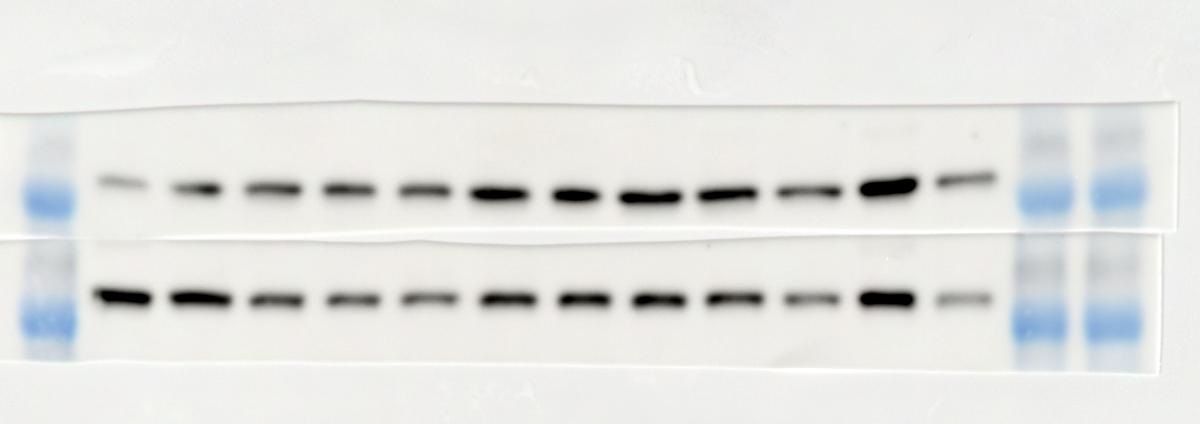

Supplement: Figure 4—source data 1. [file elife-93151-fig4-data1.zip › Figure 4 í¬ source data 1/iBAT a┴-tublin.jpg]

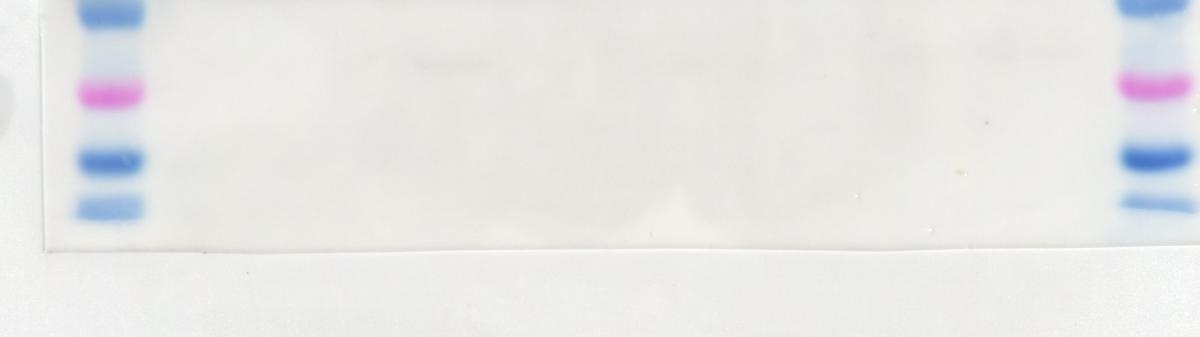

Supplement: Figure 4—source data 1. [file elife-93151-fig4-data1.zip › Figure 4 í¬ source data 1/lPRAT UCP1.jpg]

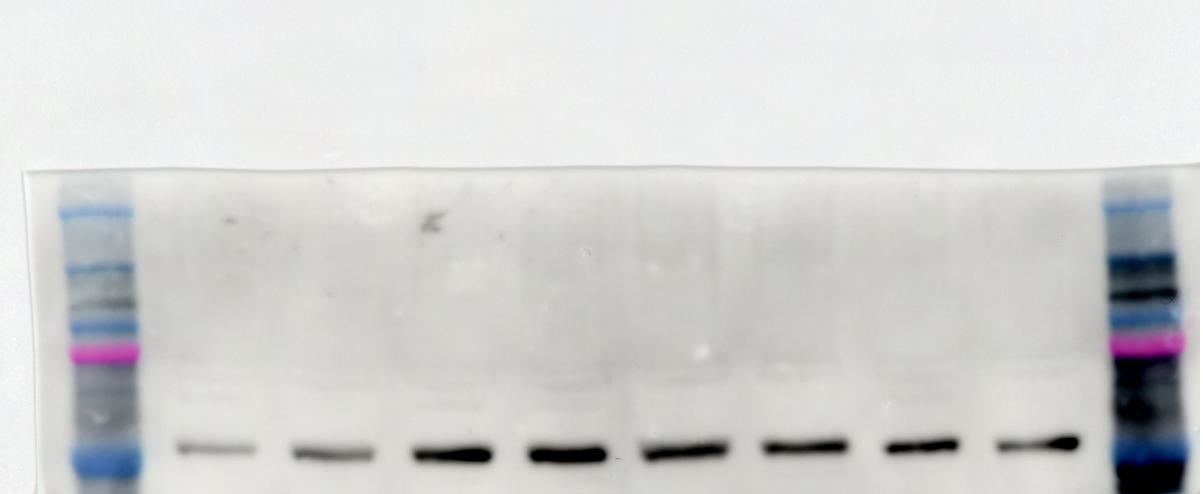

Supplement: Figure 4—source data 1. [file elife-93151-fig4-data1.zip › Figure 4 í¬ source data 1/lPRAT a┴-tublin.jpg]

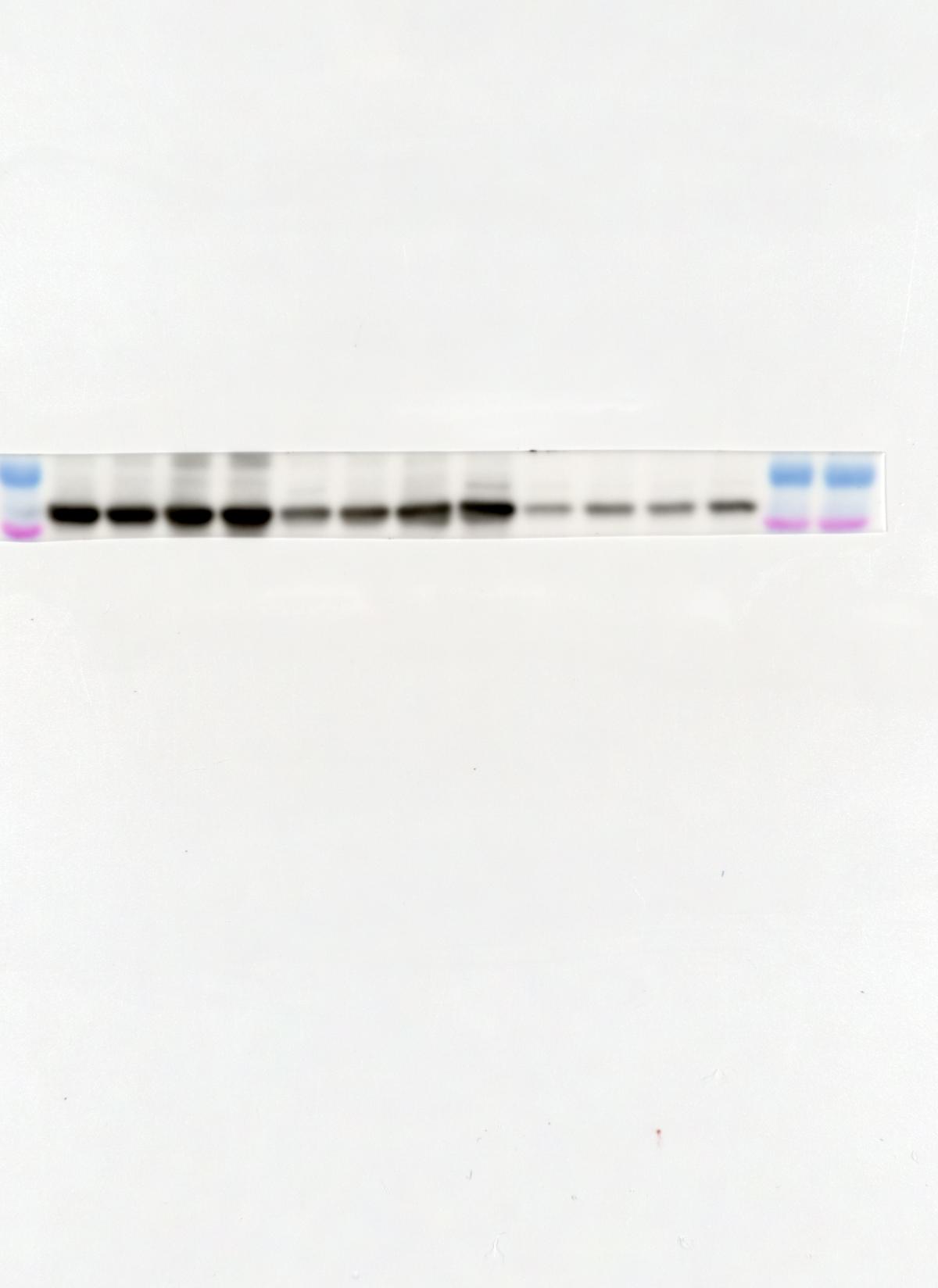

Supplement: Figure 4—source data 1. [file elife-93151-fig4-data1.zip › Figure 4 í¬ source data 1/mPRAT UCP1.jpg]

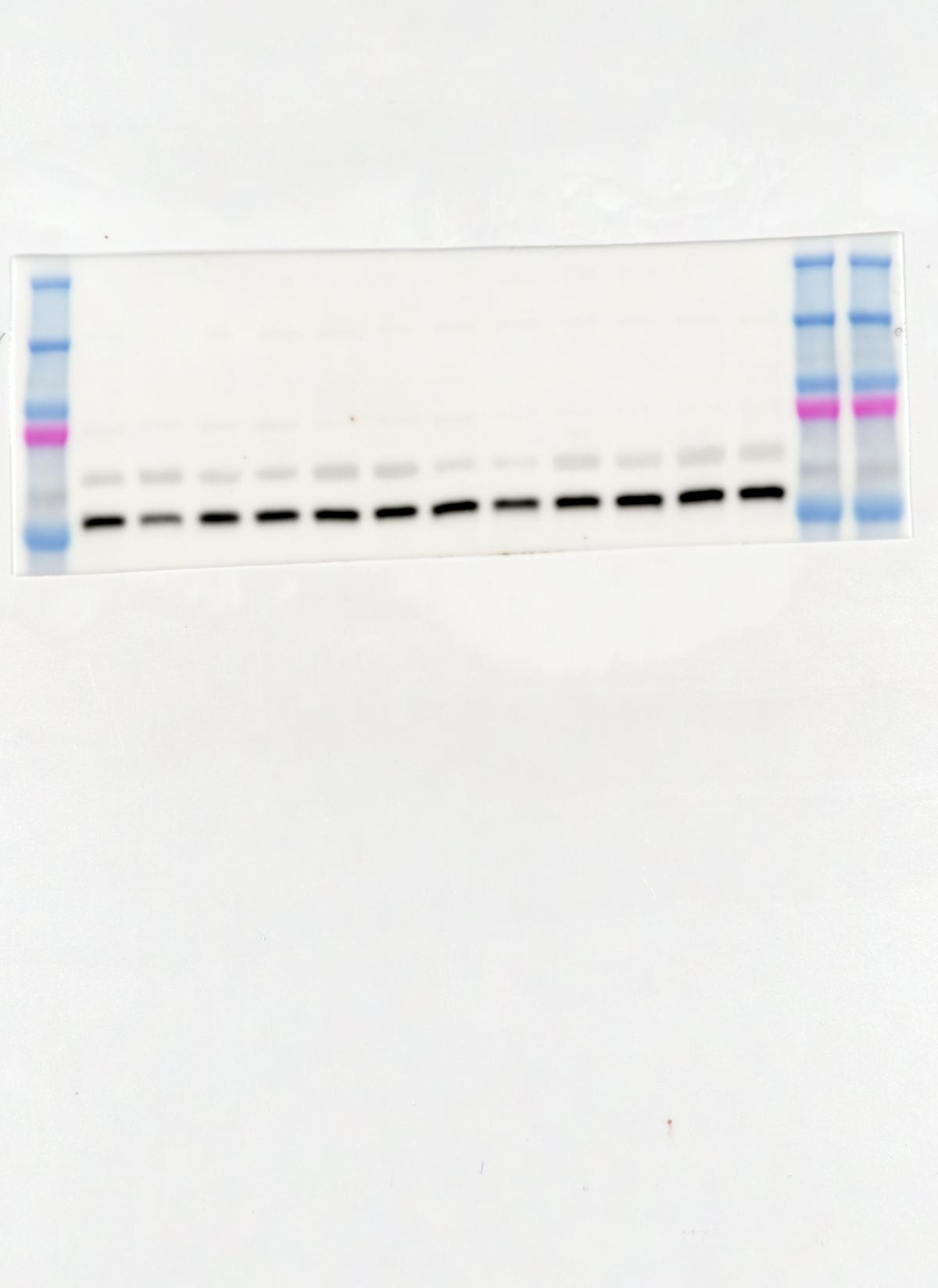

Supplement: Figure 4—source data 1. [file elife-93151-fig4-data1.zip › Figure 4 í¬ source data 1/mPRAT a┴-tublin.jpg]

Figure 4C

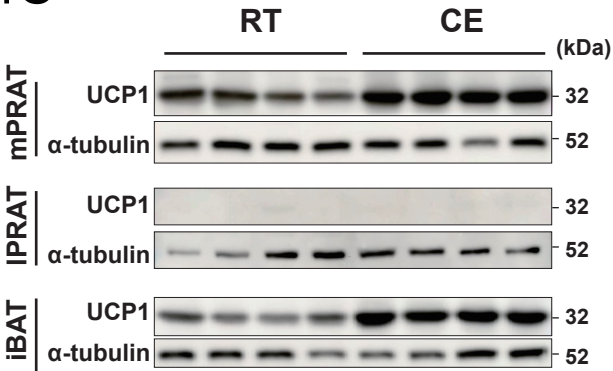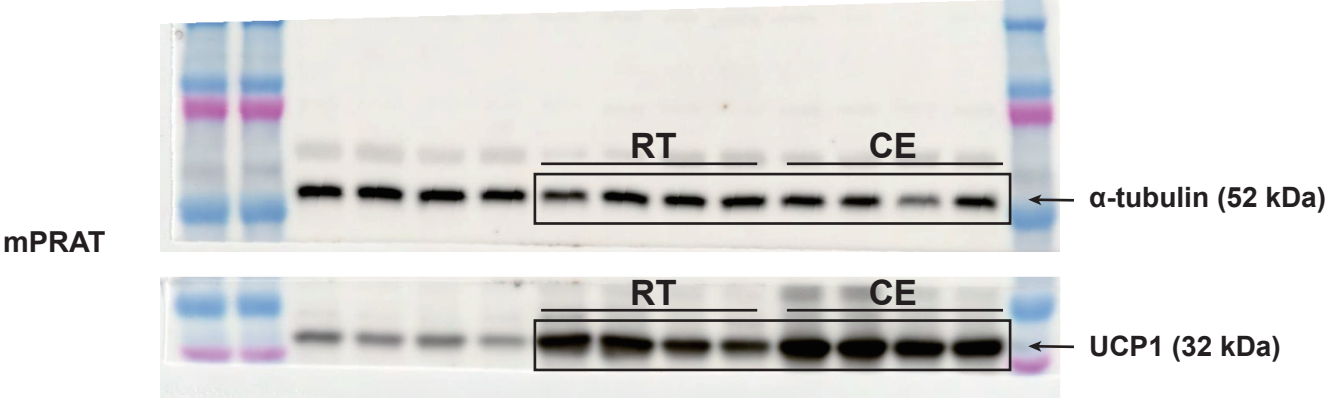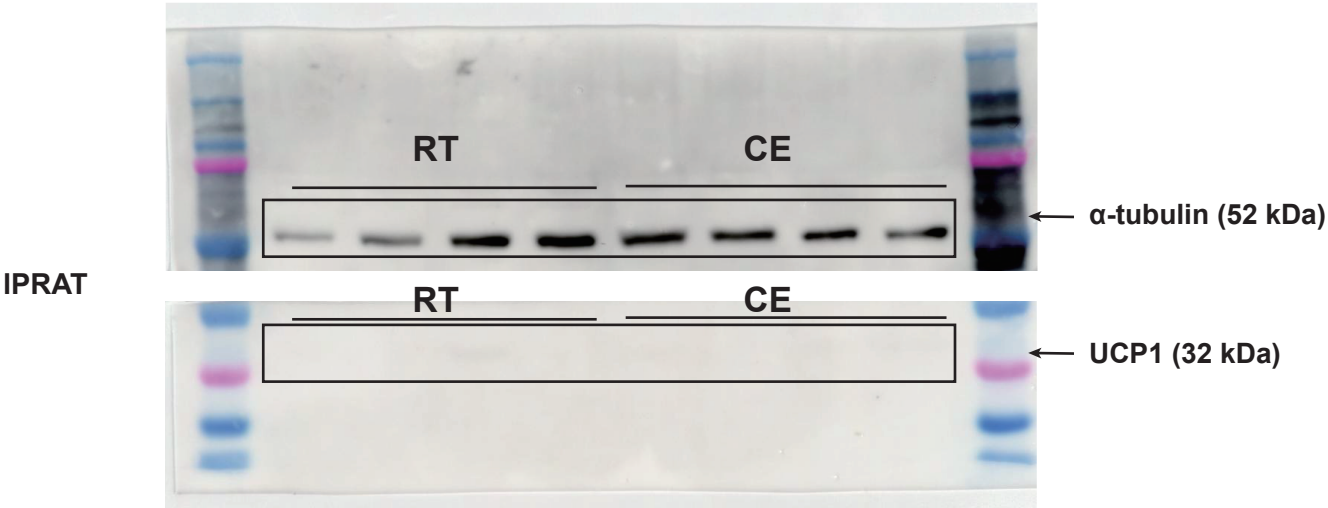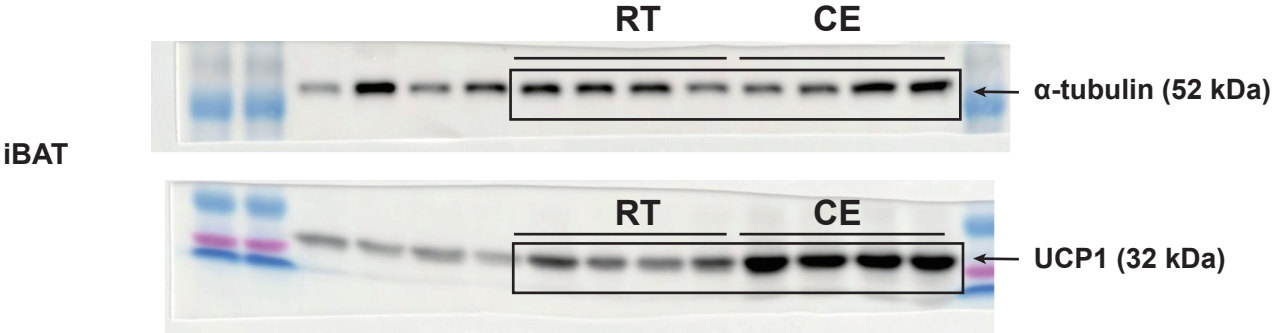

Supplement: Figure 4—source data 2. [file elife-93151-fig4-data2.zip › Figure 4 í¬ source data 2/Figure4SourceData2.pdf]
